# Co-Phosphoregulatory Network Underlying Functional Coherence of TLK1 and TLK2 Kinase Paralogs

**DOI:** 10.3390/ijms27125572

**Published:** 2026-06-20

**Authors:** Jishna Vijayan, Suhail Subair, Mukhtar Ahmed, Athira Perunelly Gopalakrishnan, Alimath Sambreena, Levin John, Rajesh Raju, Athira C. Rajeev

**Affiliations:** 1 Centre for Integrative Omics Data Science, Yenepoya (Deemed to be University), Mangalore 575018, Karnataka, India; jishnatv.ciods@yenepoya.edu.in (J.V.); suhailrejeena007@gmail.com (S.S.); athirajrf@yenepoya.edu.in (A.P.G.); alimathsambreena.ciods@yenepoya.edu.in (A.S.); 2 Department of Zoology, College of Science, King Saud University, 2455, Riyadh 11451, Saudi Arabia; mahmed1@ksu.edu.sa; 3 Institute for Regeneration and Repair, University of Edinburgh, Edinburgh EH16 4UU, UK; s2883886@ed.ac.uk

**Keywords:** Tousled-like kinases, TLK1, TLK2, phosphoproteomics, co-phosphoregulation

## Abstract

Tousled-like kinases 1 and 2 (TLK1 and TLK2) are paralogous serine/threonine kinases that share high sequence similarity yet exhibit functional divergence in cellular processes such as DNA replication, damage response, and chromatin organization. This study elucidates the paralog-specific co-phosphoregulatory networks underlying this divergence through a comprehensive analysis of 3825 human phosphoproteomic articles. Predominant phosphosites were identified as S134 and T38 for TLK1 and S73, S99, and S111 for TLK2, revealing context-dependent regulation across cancers and perturbations. Co-phosphoregulation analyses uncovered distinct networks: TLK1 associates with DNA damage signaling via proteins like ABRAXAS1, PML, and RAD9A, while TLK2 integrates with chromatin remodeling and replication through CHD4, DOT1L, NASP, and RNF20. Upstream kinases for TLK2, predominantly CDKs, link it to cell-cycle progression, whereas downstream substrates and binary interactors converge on genome stability pathways with paralog-specific nuances. These findings highlight the potential role of TLK1 on checkpoint activation and TLK2 on replication-coupled chromatin maintenance, providing insights into their roles in cancer amplification and therapeutic resistance, as well as neurodevelopmental disorders, where emerging evidence also support the involvement of TLK1 alongside TLK2.

## 1. Introduction

Tousled-like kinases 1 and 2 (TLK1 and TLK2) are paralogous serine/threonine kinases that function in several biological processes [1]. Despite their shared ancestry, TLK1 and TLK2 have diverged to regulate distinct aspects of cellular processes, including DNA repair, DNA damage response, chromatin organisation, transcription regulation, DNA replication, checkpoint control, and cell morphology [1,2]. Although TLK1 and TLK2 share similar structural features, their functional divergence is evident in their unique contributions to cell survival and development [1]. Human TLK1 and TLK2 share a similar architecture, exhibiting ~73% identity between the two. Each gene is reported to produce multiple isoforms whose functional significance remains unclear. In addition, a distinct translationally regulated variant of TLK1, known as TLK1B, has been identified and characterised [3].

Both TLK1 and TLK2 undergo extensive autophosphorylation at their N-terminal regions. This hyper-autophosphorylation limits their ability to be recruited to sites of damaged chromatin. Mechanistically, TLK1 and TLK2 are recruited via PCNA through a putative PCNA-interacting protein box (PIP-box), whose accessibility is modulated by phosphorylation of adjacent residues [1]. TLK1 and TLK2 display the highest levels of activity during the S phase of the cell cycle and are regulated by checkpoint signalling pathways activated in response to DNA damage [4,5,6]. Their most well-established function is the positive regulation of ASF1, ensuring adequate nucleosome assembly to support the increased chromatin demand during DNA replication [5,7,8].

The functional divergence of TLK1 and TLK2 as paralogs is underpinned by distinct co-phosphoregulatory networks, where their capacity to modulate the activity of the H3/H4 histone chaperone ASF1 plays a central role [9]. Beyond this shared role, TLKs have been shown to modulate kinases that control mitotic progression [10,11], participate in DNA damage-activated cell cycle checkpoint signalling, and contribute to transcriptional silencing [11,12]. TLK activity is regulated throughout the cell cycle, reaching its highest levels during S-phase progression [4,6], and becomes transiently inhibited following DNA double-strand breaks (DSBs) [13]. This inhibition in response to DNA damage is mediated by ATM and Chk1 [12]. Notably, simultaneous loss of TLK1 and TLK2 leads to marked genomic instability, reflected by elevated double-strand breaks, chromosome bridges, and other chromosomal abnormalities that are not observed when either kinase remains functional. These findings indicate that TLK1 and TLK2 perform overlapping yet paralog-specific roles in maintaining genome integrity during the DNA damage response. The TLK family comprises a distinct class of Ser/Thr kinases that are most active when cells undergo DNA replication [14]. This activity is quickly suppressed upon activation of the DNA damage response. For TLK1, this occurs through the direct phosphorylation of a C-terminal residue by checkpoint kinase 1 (CHK1) [12,13].

TLK1 has been shown to phosphorylate RAD9, a subunit of the RAD9-RAD1-HUS1 checkpoint clamp, which is essential for proper CHK1 activation, highlighting a coordinated role for TLK signalling in checkpoint control [14,15,16]. Similarly, depletion of TLK2 compromises replication fork integrity, promoting fork collapse and subsequent DNA damage accumulation [17]. Their physiological roles have been investigated through viability analyses in mouse embryos, revealing that TLK1 is dispensable during placental development, whereas TLK2 is essential for proper placental formation [18]. Recent investigations have further illuminated paralog-specific regulatory mechanisms, such as calcium overload enhancing TLK2 expression, multimerization, and phosphorylation to increase its kinase activity, forming a complex with dynein light chain LC8 and myosin IIA that drives nuclear envelope disruption and neuronal cell death in models of neurodegeneration [19]. In contrast, while both TLK1 and TLK2 exhibit redundant functions in promoting non-homologous end joining (NHEJ) repair through 53BP1 localization, their combined loss impairs NHEJ and restores homologous recombination in BRCA1-deficient cancers, conferring resistance to PARP inhibitors [20]. Additionally, in α-synucleinopathy models of Parkinson’s disease, calcium overload activates TLK2, but not TLK1, via multisite phosphorylation, suppressing autophagy and exacerbating mitochondrial and lysosomal dysfunction [21].

Both TLK1 and TLK2 are commonly amplified in various cancers, while mutations in TLK2 have been associated with neurodevelopmental disorders, including intellectual disability (ID), autism spectrum disorder (ASD), and microcephaly [9,22,23]. Emerging evidence also implicates TLK1, with recent studies reporting de novo TLK1 mutations linked to neurodevelopmental phenotypes and immunodeficiency, suggesting that both paralogs may contribute to disease [23]. Several pathogenic variants have been identified, including TLK1 mutations P25L, T38fs, and R142S [23], and TLK2 mutations D551G, E475Ter, and S617L, which are clinically associated with intellectual disability and developmental abnormalities [1,24]. Similar to other cell-cycle-associated kinases, TLKs frequently exhibit elevated kinase activity and increased gene copy number in multiple cancer types, including breast, cervical, lung, liver, colorectal, and renal malignancies [25,26]. Despite these insights into their overlapping and distinct functions, the paralog-specific co-phosphoregulatory networks that underlie the functional divergence of TLK1 and TLK2 remain incompletely characterized. In this study, we delineate these networks through phosphoproteomic data analysis, revealing key differences in substrate preferences and regulatory interactions that contribute to their specialized roles in cellular homeostasis and disease.

## 2. Results

### 2.1. Sequence Analysis of TLK1 and TLK2

**Figure 1:**
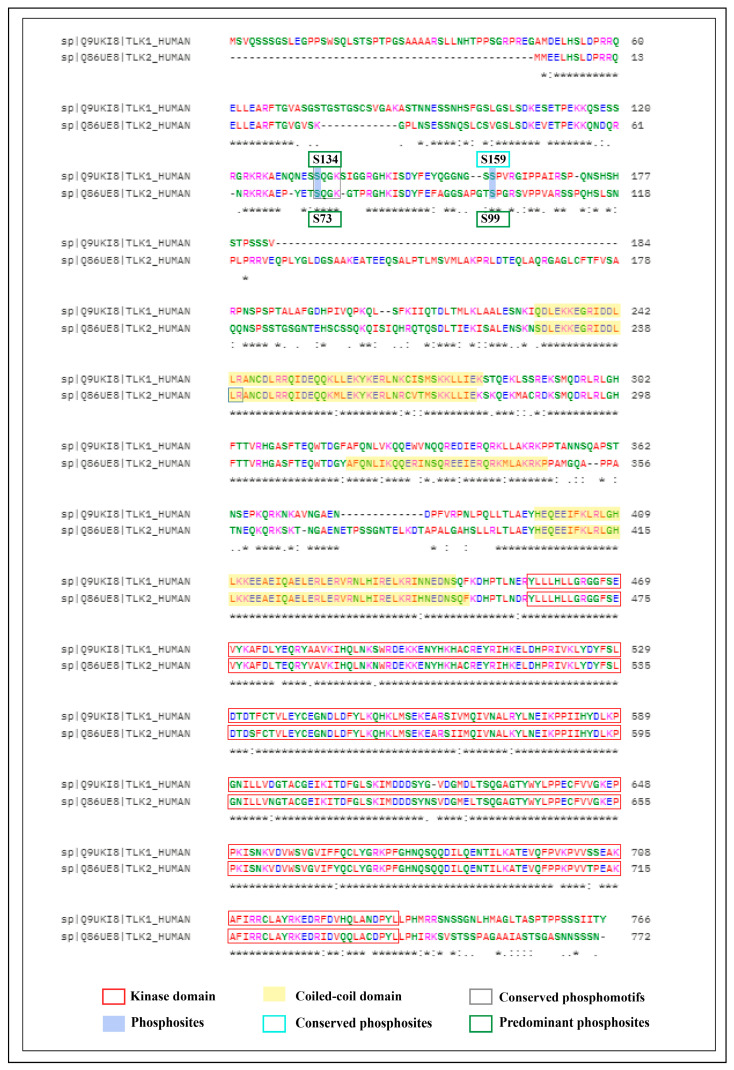
BLAST-based sequence analysis of TLK1 and TLK2 shows 73% sequence identity. Sequence alignment showing the conservation between TLK1 and TLK2, highlighting the phosphosites, predominant phosphosites, conserved phosphomotif, coiled-coil domain, kinase domain and the shared conserved phosphosite.

BLAST-based sequence analysis revealed a high degree of similarity between TLK1 and TLK2, with approximately ~73% sequence similarity (Figure 1). A very high level of conservation was observed in the C-terminal kinase domain with substantial divergence in the N-terminal regulatory regions, especially in low-complexity and phosphorylation-rich segments. Both TLK1 and TLK2 exhibited serine/threonine, proline and glycine-rich regions in the N-terminal with many SP/TP motifs likely acting as the phosphorylation sites for CDK/MAPKs. The C-terminal kinase domains are almost identical, indicating conserved enzymatic function. In contrast, their N-terminal regions are substantially divergent, enriched in low-complexity, Ser/Thr- and Pro-rich motifs that likely encode distinct regulatory inputs, phosphorylation dynamics, and interaction partners. The presence of a nuclear localization signal (NLS) in the N-terminal region is also reported in the literature [14]. Thus, the functional distinction between TLK1 and TLK2 is potentially driven by regulatory control rather than catalytic capability. These observations support the available literature that TLK1 and TLK2 can partially compensate for each other but are not fully redundant. The comparative analysis with ProtParam revealed additional variations in the physicochemical properties (Table 1).

To evaluate the evolutionary conservation of key phosphorylation sites, multiple sequence alignment of TLK1 and TLK2 orthologs from human, mouse, zebrafish, and C. elegans was performed. Protein sequences were retrieved from the UniProt database and aligned using Clustal Omega (version 1.2.4) [27]. For TLK2, C. elegans orthologous sequence was not available in UniProt and was therefore not included in the analysis. The analysis revealed differential conservation patterns across species (Supplementary Materials). For TLK1, the S134 phosphosite is conserved in human, mouse, and zebrafish, but not in C.elegans, suggesting a conserved regulatory role in higher organisms. In contrast, TLK1_T38 shows conservation only in human and mouse, indicating a more restricted evolutionary conservation. For TLK2, the S73 phosphosite is highly conserved across all examined species, highlighting its potential functional importance. However, TLK2_S99 and TLK2_S111 display conservation only in human and zebrafish, suggesting species-specific regulatory roles. Overall, these findings indicate that while some phosphosites are evolutionarily conserved, others exhibit lineage-specific patterns, reflecting both conserved and context-dependent regulatory mechanisms.

### 2.2. Class 1 Phosphosites of TLK1 and TLK2 in Global Phosphoproteome Datasets

To investigate the functionally relevant phospho-signalling patterns linked to TLK1 and TLK2, we examined a total of 3825 publicly available human phosphoproteomics articles, identifying 657 profiling and 111 differential datasets containing class 1 TLK1 phosphosites, and 641 profiling and 131 differential datasets containing class 1 TLK2 phosphosites. From these datasets, we mapped 76 and 44 phosphosites in quantitative profiles and 30 and 25 phosphosites in quantitative differential datasets for TLK1 and TLK2, respectively. The details of all qualitative and quantitative datasets used in this analysis are listed in the Supplementary File S1: Tables S1 and S2 and Supplementary File S2: Tables S1 and S2. Analysis of how these phosphosites respond to diverse experimental conditions revealed distinct regulatory patterns across cancers, infections, and various cellular perturbations for both kinases, underscoring the highly context-dependent and dynamic nature of TLK1 and TLK2 phosphorylation.

**Figure 2:**
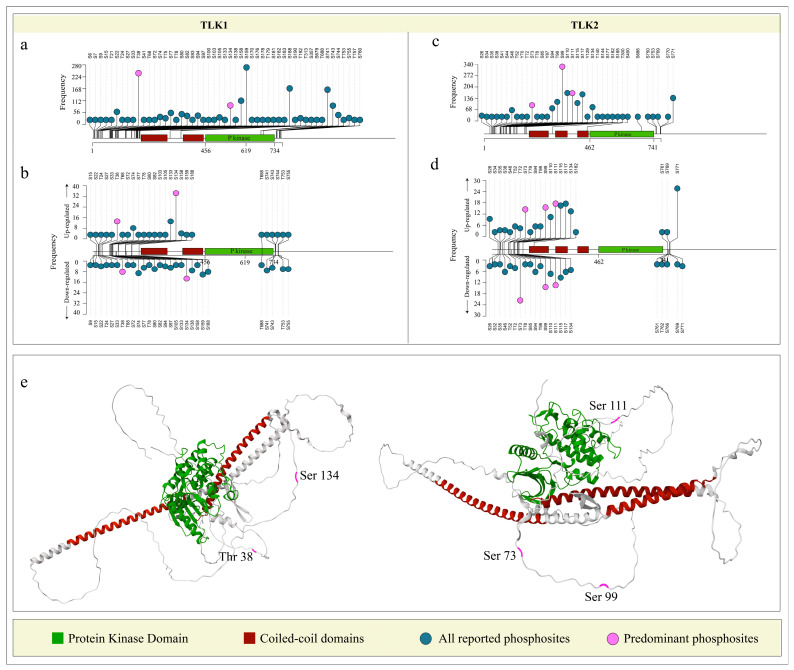
(**a**) Lollipop plot illustrating the phosphorylation sites of TLK1 identified in profiling datasets. (**b**) Lollipop plot showing phosphorylation sites of TLK1 from differential datasets. (**c**) Lollipop plot illustrating the phosphorylation sites of TLK2 identified in profiling datasets. (**d**) Lollipop plot showing phosphorylation sites of TLK2 from differential datasets. (**e**) Structural model of TLK1 and TLK2 highlighting the location of the predominant phosphosites. The protein kinase domain is shown in green, coiled-coil domain in red, while the rest of the structure is represented in gray. The key phosphorylation sites are indicated in pink and labelled accordingly.

We ranked all TLK1 and TLK2 phosphosites based on their detection frequency across publicly available articles. For TLK1, S134 and T38 emerged as the most frequently detected and differentially regulated phosphosites. While S73, S99, and S111 of TLK2 were identified as the predominant phosphosites, they also exhibited differential regulation across conditions. (Figure 2a–d). Since experimentally determined full-length structures of TLK1 and TLK2 are not available, we used AlphaFold-predicted structures (UniProt IDs: Q9UK18 for TLK1 and Q86UE8 for TLK2) to perform structural mapping (Figure 2e). This allowed us to visualize the positions of the predominant phosphosites relative to the kinase domain and other regions in both proteins. The predominant phosphosites, Ser134 and Thr38 in TLK1, and Ser73, Ser99, and Ser111 in TLK2, were observed to be situated outside the kinase domain (pink) in the N-terminal regions of both proteins. This distribution indicates the possibility that regulation of TLK1 and TLK2 primarily occurs through phosphorylation of their non-catalytic domains.

### 2.3. High-Confidence Proteins Co-Phospho-Regulated with Predominant TLK1 and TLK2 Phosphosites

**Figure 3:**
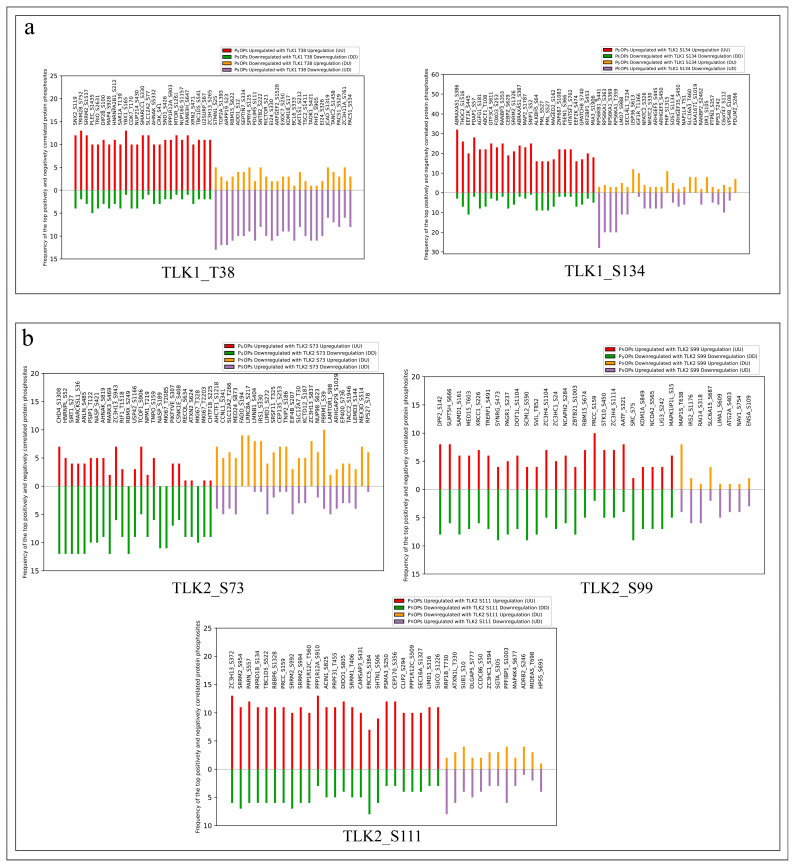
(**a**) Top 25 high-confidence proteins showing positively co-phosphoregulated and negatively co-phosphoregulated PsOPs of predominant TLK1 phosphosites. (**b**) Top 25 high-confidence proteins showing positively co-phosphoregulated and negatively co-phosphoregulated PsOPs of predominant TLK2 phosphosites.

Following the mapping of the primary phosphorylation sites, we performed co-phosphoregulation analyses to pinpoint high-confidence phosphosites that demonstrate coordinated modulation with TLK1 and TLK2 (Supplementary File S1: Tables S3–S6 and Supplementary File S2: Tables S3–S8). Through Fisher’s Exact Test (FET) analysis, we determined that the TLK1_S134 site displayed positive co-phosphoregulation with 795 phosphosites in other proteins (PsOPs) and negative co-phosphoregulation with 29 PsOPs. The TLK1_T38 site showed positive co-phosphoregulation with 290 PsOPs and negative co-phosphoregulation with 258 PsOPs. Conversely, TLK2_S73 exhibited positive co-phosphoregulation with 161 PsOPs and negative co-phosphoregulation with 53 PsOPs. For the S99 site, 378 phosphosites demonstrated positive co-phosphoregulation, while 8 showed negative co-phosphoregulation. At the S111 site, 753 phosphosites were positively co-phosphoregulated, and 12 were negatively co-phosphoregulated. The expression patterns of a few of these high-confidence proteins are depicted in Figure 3.

Among these, BRCA1-A complex subunit Abraxas 1/ABRAXAS1_S386 was positively co-phosphoregulated with the TLK1_S134 site. ABRAXAS1 serves as a key element of the BRCA1-A complex, which detects ubiquitinated chromatin at DNA double-strand breaks (DSBs). In response to DNA damage, ABRAXAS1 facilitates the recruitment of BRCA1 to DSB sites, promoting effective activation of DNA damage response (DDR) pathways, such as checkpoint signaling and repair orchestration [28]. Promyelocytic Leukemia protein/PML_S527 also exhibited positive co-phosphoregulation with TLK1_S134. PML functions as a vital tumor suppressor that assembles PML nuclear bodies (PML-NBs), serving as scaffolds for DNA damage response (DDR) signaling [29]. Additionally, Cyclin-Dependent Kinase 7/CDK7_T170 showed positive co-phosphoregulation with TLK1_T38. CDK7 is critical for preserving genome stability and holds a significant position in regulating gene transcription, especially in the context of cellular responses to DNA damage [30].

The DNA damage response and chromatin regulatory network proteins like CHD4_S1308, RIF1_T1518, NASP_S421, and SIRT1_S27 displayed positive co-phosphoregulation with the TLK2_S73 site and are connected functionally to TLK2 via their contributions to histone H3-H4 stabilization (NASP), chromatin remodeling (CHD4), checkpoint regulation (RIF1), and genome integrity (SIRT1). Phosphorylation at the TLK2_S99 site was linked to proteins central to DNA damage signaling and repair, such as DOT1L_S1104, DPF2_S142, and AATF_S321, each showing positive co-phosphoregulation with TLK2_S99. DOT1L, found within the TLK2 co-phosphoregulated network, acts as a chromatin-linked regulator involved in transcriptional oversight and DNA repair, with recognized functions in development, cell-cycle advancement, and genome preservation [31]. The inclusion of DPF2_S142 additionally highlights TLK2’s role as a coordinator of chromatin dynamics, DNA repair, and cell cycle control. Furthermore, AATF_S321, a factor responsive to stress, aids in safeguarding cells from various stressors, including DNA damage [32], thereby reinforcing the connection between TLK2_S99 phosphorylation and mechanisms of genome stability.

### 2.4. Predicted Upstream Kinases of TLK2

To investigate the molecular networks connected to TLK1 and TLK2, we examined upstream kinases exhibiting co-phosphoregulation at specific phosphosites. We identified a total of 14 upstream kinases that were positively co-phosphoregulated with both TLK2_S99 and TLK2_S111 (Supplementary File S2: Table S9). Our dataset did not uncover any experimentally confirmed or predicted upstream kinases for TLK1.

**Figure 4:**
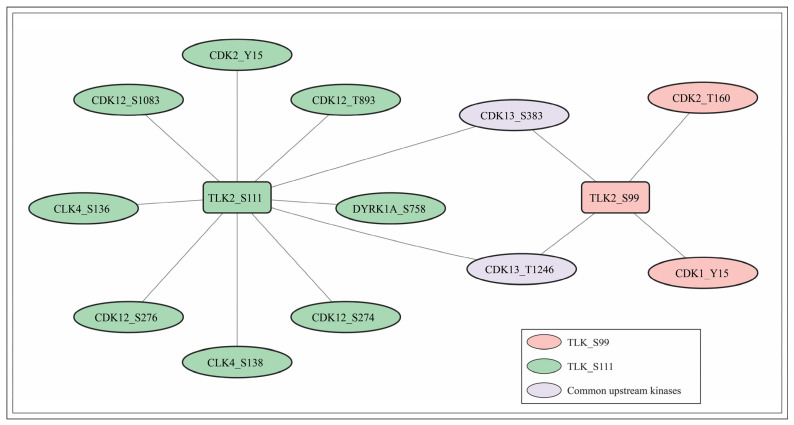
Predicted upstream kinases among the co-phosphoregulatory networks of key phosphosites of TLK2.

In our data, CDK1_Y15, CDK2 (T160, Y15), CDK12 (T893, S1083, S274, S276), CDK13 (S383, T1246), DYRK1A_S758, and CLK4 (S136, S138) demonstrate positive co-phosphoregulation with TLK2_S99 and TLK2_S111 (Figure 4). Cyclin-dependent kinases (CDKs) serve as key controllers of fundamental cellular functions. By phosphorylating targeted substrates, they oversee cell-cycle advancement, including aspects like cell proliferation, DNA synthesis, and additionally influence transcription, metabolic processes, and DNA repair mechanisms. Additionally, this group encompasses standard regulators of cell-cycle advancement and checkpoint activation, including CDK1, CDK2, CDK12, and CDK13, which function as cyclin-dependent kinases vital for the G1/S and G2/M phases [33]. CDK1, CDK2, and CDK4 directly manage cell-cycle phase shifts and cellular division [34]. CDK12 is pivotal for preserving genome stability through its control of transcription for essential DNA damage response (DDR) genes [35]. Disruption of CDK12 function hinders accurate transcription of DDR genes, resulting in weakened genomic upkeep and eventually fostering genome instability, a hallmark of numerous human malignancies [36]. Dual specificity tyrosine phosphorylation-regulated kinase 1A (DYRK1A) phosphorylates an extensive array of proteins that govern transcriptional pathways, cell-cycle dynamics, DNA damage repair, apoptosis signaling, and cytoskeletal structure [37]. CDK13 (S383, T1246) and CDK2 (T160, Y15) stood out as shared upstream kinases for both TLK2_S99 and TLK2_S111, suggesting that these phosphorylation sites are governed by overlapping CDK-mediated signaling routes. This commonality implies synchronized oversight of TLK2 function, possibly connecting its regulation to cell-cycle progression and transcriptional mechanisms directed by CDKs.

The notable abundance of cyclin-dependent kinases and checkpoint regulators highlights a close functional link between TLK2 activity, cell-cycle progression, transcriptional regulation, and genome stability. These findings suggest that TLK2 probably plays a role in key cell-cycle and checkpoint signaling pathways. They also emphasize the potentials of coordinated regulation by upstream kinases to fine-tune TLK2 activity according to cell-cycle demands and DNA damage conditions.

Interestingly, the phosphosites TLK2_S99 and TLK2_S111 are predicted to share several upstream kinases, including CDK13 and CDK2. This suggests that these sites may be regulated by overlapping mechanisms, allowing coordinated control of TLK2 function in response to cell-cycle signals and DNA damage.

### 2.5. Downstream Substrates Among the Co-Phospho-Regulated Network of TLK1 and TLK2

**Figure 5:**
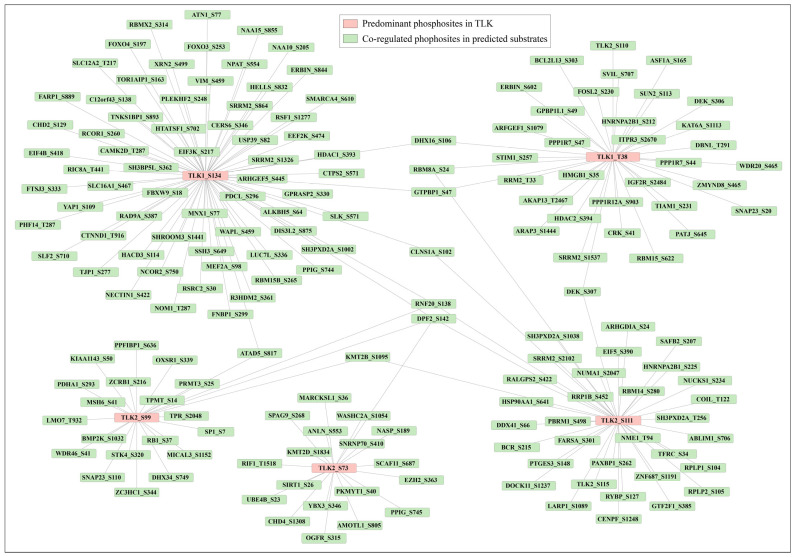
Downstream substrates of TLK1 and TLK2 among the co-phosphoregulatory network.

For TLK1_S134, phosphorylation showed positive co-phosphoregulation with 74 substrates and negative co-phosphoregulation with one substrate (Figure 5) (Supplementary File S1: Table S7). Key examples include RAD9 checkpoint clamp component A (RAD9A_S387), an essential protein central to the DNA damage response, and ATPase Family AAA Domain Containing 5 (ATAD5_S817), which plays a vital role in DNA replication and repair.

The TLK1_T38 site exhibited positive co-phosphoregulation with 26 substrates and negative co-phosphoregulation with 13 substrates (Supplementary File S1: Table S8). Notable among these are the anti-silencing function 1 A histone chaperone (ASF1A_S165), a core histone chaperone that binds H3-H4 to facilitate nucleosome assembly during DNA replication and repair [38], and TLK2_S110, a protein kinase involved in DNA replication, DNA repair, and cell cycle control.

In contrast, TLK2_S73 phosphorylation was positively co-phosphoregulated with 16 substrates and negatively co-phosphoregulated with three (Supplementary File S2: Table S10). A prominent substrate here is Nuclear Autoantigenic Sperm Protein (NASP_S189), which is required for DNA replication and cell cycle progression. For TLK2_S99, 23 substrates displayed positive co-phosphoregulation (Supplementary File S2: Table S11), including ATAD5_S817, a PCNA unloader that maintains replication fork stability and supports chromatin restoration after DNA damage, highlighting mechanistic overlap with TLK2’s roles in S-phase progression and genome stability.

Phosphorylation at TLK2_S111 showed positive co-phosphoregulation with 38 substrates (Supplementary File S2: Table S12), with RNF20_S138, DEK_S307, and NUCKS1_S234 standing out for their functional alignment with TLK2. Ring Finger Protein 20 (RNF20) regulates chromatin accessibility at DNA damage sites via H2B ubiquitination [39], complementing TLK2’s involvement in histone supply and chromatin assembly during replication and repair. DEK, a chromatin-associated factor, is closely tied to replication stress responses and genome integrity [40], mirroring TLK2’s function in replication-coupled chromatin restoration. Additionally, Nuclear Ubiquitous Casein and Cyclin-Dependent Kinase Substrate 1 (NUCKS1) modulates chromatin architecture, DNA repair, and cell cycle regulation, reinforcing its connection to TLK2 in checkpoint recovery post-DNA damage [41].

Notably, DPF2_S142 emerged as a shared downstream substrate across TLK1_S134, TLK2_S73, TLK2_S99, and TLK2_S111, implying coordinated regulation among these TLK phosphosites. Similarly, RNF20_S138 was commonly targeted by TLK1_S134, TLK2_S99, and TLK2_S111, reflecting a convergence of TLK1 and TLK2 signaling pathways on this substrate. Additional overlaps included RNA-binding motif protein 8A (RBM8A_S24) and protein DEK (DEK_S307) as common substrates of TLK1_T38 and TLK2_S111; GTP-binding protein 1 (GTPBP1_S47) and DEAH-box helicase 16 (DHX16_S106) shared between TLK1_T38 and TLK1_S134; chloride nucleotide-sensitive channel 1A (CLNS1A_S102) linked to TLK1_S134 and TLK2_S111; ATPase family AAA domain-containing 5 (ATAD5_S817) associated with TLK1_S134 and TLK2_S99; and lysine methyltransferase 2B (KMT2B_S1095) targeted by TLK2_S99 and TLK2_S111. These recurrent shared substrates across distinct TLK1 and TLK2 phosphosites underscore a framework of coordinated and convergent signaling, emphasizing their synergistic contributions to genome integrity and gene regulation.

Overall, the analysis of downstream substrates in the co-phosphoregulated networks of TLK1 and TLK2 predicts convergence on key pathways involved in DNA replication, chromatin assembly, and genome stability. A notable shared substrate is DPF2_S142 (Double PHD Fingers 2), a subunit of the BAF (mSWI/SNF) chromatin remodeling complex. DPF2 functions as a chromatin-recognition component by binding to modified histone tails, thereby potentially guiding the complex’s remodeling activity. Its positive co-phosphoregulation with TLK1_S134, TLK2_S73, TLK2_S99, and TLK2_S111 points to coordinated regulation across these TLK phosphorylation sites.

### 2.6. Predicted Binary Interactors of TLK1 and TLK2

**Figure 6:**
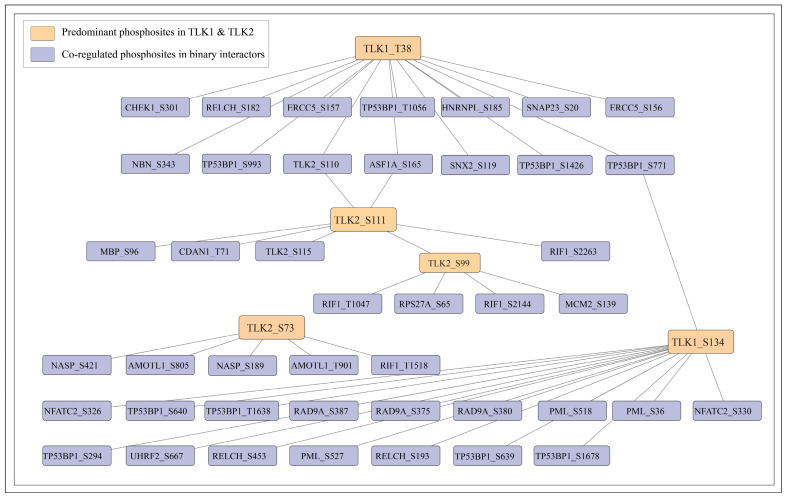
Illustration of predicted binary interactors of TLK1 and TLK2 at major phosphosites is shown.

TLK1_S134 was identified to be associated with 19 co-phosphoregulated binary interactors (Figure 6) (Supplementary File S1: Table S9). Notably, RAD9 checkpoint clamp component A/RAD9A_S380 showed positive co-phosphoregulation with this site. RAD9A plays a critical role in the cellular DNA damage response and directly influences DNA repair [42,43], potentially regulating DNA double-strand break repair through its interactions with checkpoint signaling components [44]. Additionally, tumor protein p53 binding protein 1 (TP53BP1), phosphoregulated at multiple sites (S639, S771, S640, T1638, S294, S1678), exhibited positive co-phosphoregulation with TLK1_S134. TP53BP1 is involved in TP53-dependent transcriptional activation and DNA damage-induced checkpoint signaling [45].

TLK1_T38 was linked to 14 co-phosphoregulated binary interactors (Supplementary File S1: Table S10). Key positive co-phosphoregulations included ASF1A_S165 and NBN_S343. The anti-silencing function 1 A histone chaperone (ASF1A) binds histones H3 and H4, collaborating with chromatin assembly factors to promote nucleosome assembly during DNA replication and repair [17,46]. Nibrin (NBN) contributes to DNA repair and the broader DNA damage response [47]. TP53BP1 showed both positive and negative co-phosphoregulation with TLK1_T38, acting as a central mediator in the DNA damage response pathway to coordinate signaling and repair after DNA damage [48]. In contrast, CHEK1_S301 displayed negative co-phosphoregulation with TLK1_T38. Checkpoint kinase 1 (CHEK1) is a pivotal regulator of DNA replication, mitotic progression, DNA repair, and cell cycle coordination [49].

For TLK2_S73, five binary interactors exhibited co-phosphoregulation. Among them, NASP_S189 showed positive co-phosphoregulation. Nuclear Autoantigenic Sperm Protein (NASP) supports DNA repair processes [50] and protects soluble H3-H4 from degradation, potentially functioning as a histone chaperone during DNA damage [51]. TLK2_S99 was associated with five co-phosphoregulated binary interactors, including MCM2_S139, which displayed positive co-phosphoregulation. Minichromosome maintenance complex component 2 (MCM2) is essential for regulating DNA replication [52].

Seven binary interactors were co-phosphoregulated with TLK2_S111, with ASF1A_S165 and MBP_S96 showing positive co-phosphoregulation. ASF1A, a primary substrate of TLKs, undergoes phosphorylation that enhances nucleosome assembly during DNA replication [17], enabling TLK2 to promote cell cycle progression by increasing histone availability [53]. Myelin basic protein (MBP), a major component of central nervous system myelin [54], directly interacts with and is phosphorylated by TLK2, often serving as a substrate for assessing TLK2 kinase activity [14] (Supplementary File S2: Table S13).

Collectively, these co-phosphoregulation analyses reinforce TLK1 and TLK2 as crucial coordinators that integrate DNA damage signaling, chromatin organization, and replication-related functions.

## 3. Discussion

In this study, we have delineated the paralog-specific co-phosphoregulatory networks of TLK1 and TLK2 through a comprehensive analysis of phosphoproteomic datasets, revealing distinct phosphorylation patterns and associated regulatory interactions that underpin their functional divergence. Despite sharing ~73% sequence similarity, particularly in their C-terminal kinase domains, TLK1 and TLK2 exhibit substantial differences in their N-terminal regulatory regions, which are enriched in low-complexity motifs and phosphorylation sites. These structural variations likely contribute to differential regulatory inputs, enabling specialized roles in cellular processes such as DNA replication, damage response, and chromatin organization. Our findings align with prior reports indicating that while TLK1 and TLK2 can partially compensate for each other, their non-redundant functions are evident in contexts such as genome maintenance and DNA repair [9]. Recent cellular and molecular studies have shown that the loss or reduction in TLK1 and/or TLK2 function can confer resistance to PARP inhibitors (PARPi) in BRCA1-deficient cancer models by impairing NHEJ and altering 53BP1 recruitment [20]. TLK2 has a specific essential function in placental development in mouse models that TLK1 does not compensate for: TLK2-deficient embryos die due to placental failure, while TLK1 deletion does not produce this phenotype [9].

We identified several predominant phosphosites: S134 and T38 for TLK1, and S73, S99, and S111 for TLK2. These sites were frequently detected and showed differential regulation across various conditions, including different cancers and cellular perturbations, highlighting their functional importance. For example, the positive co-phosphoregulation of TLK1_S134 with ABRAXAS1_S386 and PML_S527 suggests that TLK1 may integrate into BRCA1-mediated DNA damage response pathways [18,55] and PML nuclear body functions, which are important for checkpoint activation and tumor suppression [56,57]. Similarly, TLK2’s main phosphosites co-phosphoregulate with proteins such as CHD4_S1308, RIF1_T1518, and DOT1L_S1104, supporting its involvement in chromatin remodeling [58], replication fork stability, and transcriptional regulation during S-phase [1,17,59].

Comparative sequence analysis indicates that some of the predominant phosphosites are evolutionary conserved, while others display more restricted conservation patterns. This suggests that certain phosphorylation events are preserved across evolution and may play fundamental roles, whereas others are likely to be context-dependent and contribute to paralog-specific functional diversification. The known functional roles of the reported phosphosites of both TLK1 and TLK2 is very limited. Few Phosphosites like S743 is reported to have enzyme inhibitory role [60] as and TLK1_S134 has been reported as a hyperphosphorylated residue and is suggested to function as an autophosphorylation site. N-terminal autophosphorylation of TLK1 regulates its recruitment to damaged chromatin by modulating PCNA interaction, with increased phosphorylation limiting chromatin association during the DNA damage response. These findings provide functional context for the observed co-phosphoregulation patterns and support a role for S134 in modulating TLK1-mediated checkpoint signaling [1].

Analysis of upstream kinases showed that TLK2 is mainly linked to cyclin-dependent kinases (for example, CDK1_Y15, CDK2_T160, and CDK12_T893), which are positively co-phosphoregulated with S99 and S111. This suggests that TLK2 acts as a downstream effector in cell-cycle progression and transcriptional control, in line with its known peak activity during S-phase and transient inhibition after DNA damage via CHK1 [13,33]. In contrast, no upstream kinases were clearly identified for TLK1 in our dataset, which may be due to limitations in the data or could indicate different regulatory mechanisms, such as autophosphorylation or calcium-dependent pathways reported for TLK2 in certain contexts [21].

Downstream substrates and interacting proteins in these networks converge on DNA replication and repair, but also show paralog-specific features. For TLK1, substrates such as RAD9A_S387 and ASF1A_S165 link it to checkpoint signaling [61,62] and nucleosome assembly, consistent with its role in RAD9 phosphorylation and CHK1 activation [5,13,53]. For TLK2, substrates including NASP_S189, RNF20_S138, and DEK_S307 point to roles in histone chaperoning, ubiquitination, and replication stress responses, aligning with its importance in preventing replication fork collapse [9,59,63]. Notably, DPF2_S142 showed positive co-phosphoregulation across multiple sites in both TLK1 and TLK2, suggesting a shared connection with BAF chromatin remodeling complexes [64] that may help regulate histone tail recognition and chromatin remodeling during DNA repair [65]. Binary interaction data further support specialization: TLK1 is associated with multisite phosphorylation of TP53BP1, which promotes 53BP1-dependent NHEJ [20], while TLK2 co-phosphoregulates with replication licensing factors such as MCM2_S139, consistent with its role in replication-coupled chromatin maintenance during S-phase [59]. Together, these patterns indicate that TLK1 is more involved in damage signaling, whereas TLK2 contributes more to chromatin dynamics during replication.

These findings have potential implications for disease. Amplifications of TLK1 and TLK2 in cancers, as well as TLK2 mutations in neurodevelopmental disorders, suggest that targeting paralog-specific networks could have therapeutic value. In addition, de novo mutations in TLK1 have been reported in association with neurodevelopmental phenotypes and immunodeficiency, supporting a role for both paralogs in disease pathogenesis. For instance, interfering with TLK2’s CDK-regulated sites might sensitize BRCA1-deficient tumors to PARP inhibitors by impairing NHEJ. However, our study has limitations, including reliance on public datasets (which may be biased toward certain conditions) and the lack of raw data re-analysis. In addition, while in silico predictions are useful, experimental validation through site-specific studies in relevant cellular models is still needed.

In summary, this phosphoproteomic analysis highlights paralog-specific co-phosphoregulatory networks that contribute to the functional divergence of TLK1 and TLK2, mainly in DNA damage response and chromatin homeostasis. These insights may support the development of targeted approaches for cancer and neurodevelopmental disorders. Future work involving site-specific mutagenesis and high-resolution interactomics will help validate these mechanisms.

## 4. Materials and Methods

### 4.1. TLK1 and TLK2 Sequence Analysis

The protein sequences of TLK1 (Uniprot: Q9UKI8) and TLK2 (Uniprot: Q86UE8) were retrieved for comparative analysis of their sequence composition. Pairwise sequence analysis was determined using BLAST (version 2.17.0) [66], while conserved regions and domain architecture were validated through multiple sequence alignment performed with Clustal Omega [27]. Key physicochemical properties such as molecular mass, predicted isoelectric point (pI), and protein stability (instability index) were computed using the ExPASy ProtParam tool [67].

### 4.2. Data Curation and Identification of Key Sites in TLK1 and TLK2

A comprehensive search was conducted on PubMed using the keywords “Phosphoproteomics” or “Phosphoproteome”, with an explicit exclusion of plant-related studies and review articles. From this search, we compiled datasets specifically of the human cellular phosphoproteome that reported Class 1 phosphosites on the TLK1 and TLK2 kinases. Class 1 sites were identified by a localisation probability greater than 75% and an A score exceeding 13, ensuring a high level of confidence in site assignment. The primary objective of this study was to pinpoint the most frequently modified phosphorylation sites of TLK1 and TLK2, specifically on serine, threonine, and tyrosine residues. The curated datasets were grouped into two categories: profile datasets, which indicate the presence of phosphosites, and differential datasets, which track quantitative changes across varying experimental conditions. For the differential datasets, the focus was on regulated Class 1 phosphosites [68].

Phosphorylation sites were classified as upregulated if the fold change was ≥1.3 and downregulated if ≤0.76, provided these changes were statistically significant (*p* < 0.05) based on the criteria outlined in the original publications. These rigorous parameters were applied to ensure that only biologically relevant phosphorylation changes were considered. To ensure consistent protein and phosphosite annotations, we applied a custom mapping pipeline that aligned protein identities to standardised human gene symbols using the HGNC database [69], and the phosphosite mapping was based on the Uniprot database [70]. A custom-built tool was employed to extract and align phosphosite data, including the modified residue, its position, and the surrounding sequence, mapped to UniProt accessions with isoform-level precision. Integrated validation steps ensured reliable annotation.

Class 1 phosphosites of TLK1 and TLK2 were identified from human phosphoproteome datasets, and their occurrence was analysed across both quantitative and qualitative profiles. Sites detected in at least 50% of both dataset types were classified as predominant. Phosphosites that were not frequently detected/not reported as Class-1 sites within the phosphoproteome datasets were excluded from the analysis.

### 4.3. Structural Mapping and Domain Annotation

To interpret the identified phosphorylation sites within structural and functional regions, an integrated sequence-structure mapping approach was employed. Phosphosites derived from curated datasets were mapped onto the full length TLK1 and TLK2 protein sequences and visualized using lollipop plots to depict their positional distribution. Three dimensional structural domains were obtained from the AlphaFold protein structure database [71] and used to examine the spatial localisation of phosphorylation sites. Domain architecture, including kinase domains and coiled-coil regions, was annotated based on UniProt.

### 4.4. Filtering the Co-Phospho-Regulated Proteins of Predominant TLK1 and TLK2 Phosphosites

To identify phosphosites in other proteins (PsOPs) that are positively or negatively co-phosphoregulated with the predominant phosphosites of TLK1 (pS134 and pT38) and TLK2 (pS73, pS99, and pS111), the quantitative profile datasets corresponding to each TLK1 and TLK2 site were analysed individually. Given the large number of datasets covering diverse biological systems, experimental conditions, and analytical platforms, re-analysis of raw data was not feasible. Therefore, only phosphosites with a localisation probability ≥75% and an A-score ≥ 13 were included. Each quantitative profile dataset, regardless of potential bias from the over-representation of specific conditions in test-versus-control comparisons, was grouped into two categories: (category 1) datasets where a given TLK1 or TLK2 phosphosite was upregulated (U), and (category 2) datasets where it was downregulated (D). From these categories, PsOPs were identified based on their differential behaviour relative to the TLK phosphosite: those upregulated or downregulated when a TLK site was upregulated (UU and UD, respectively) and those upregulated or downregulated when a TLK site was downregulated (DU and DD, respectively) [72].

We quantified how many quantitative profile datasets showed each PsOP forming a specific relationship with the predominant TLK1 and TLK2 phosphosites across the UU, DD, UD, and DU categories. We assumed that PsOPs in the UU and DD groups reflect positive co-phosphoregulation with TLK phosphosites, while those in the UD and DU groups indicate negative co-phosphoregulation. Based on this, PsOPs were categorised into UUDD (PsOPs that increase or decrease in parallel with TLK phosphosites) and UDDU (PsOPs that increase when TLK phosphosites decrease, or vice versa). The UUDD group represents strong positive co-phosphoregulation, whereas the UDDU group reflects strong negative co-phosphoregulation [73]. To evaluate co-phosphoregulation between predominant TLK1 and TLK2 phosphosites and their associated PsOPs, we applied a one-sided Fisher’s exact test (FET). PsOPs with *p* < 0.05 in the UUDD (positive) or UDDU (negative) categories were considered significant. Differential FET scores were calculated using all available differential datasets, and a high confidence cutoff of 10% of the total differential datasets for each TLK phosphosites was applied. High confidence TLK phosphosite PsOP pairs were those that met three criteria: FET *p* < 0.05, a ratio exceeding the 10% threshold, and detection across at least three studies and three distinct experimental conditions. These pairs were then used for analysis of biological functions, pathways, protein interactions, and kinase-substrate relationships, ensuring robust and unbiased phosphosite associations.

### 4.5. Examination of Upstream Kinase, Downstream Substrates and Binary Interactors

Experimentally validated upstream kinases and protein substrates associated with TLK1 and TLK2 were compiled from curated databases, including PhosphoSitePlus [74], Phospho.ELM 9.0 [75], RegPhos 2.0 [76]. In addition, TLK1 and TLK2 specific upstream kinases and substrates were predicted using multiple in silico tools, namely NetworKIN [77], AKID [78], and iKip-DB [79]. Additionally, datasets of TLK1 and TLK2 substrates identified by Johnson et al. (2023) [80] through a synthetic peptide library-based screening approach were retrieved, and datasets with a ≥90% confidence cutoff were included in the current study.

Protein–protein interactions were compiled from several curated databases, including HPRD [81], BIND [82], BioGRID [83], and ConsensusPathDb (version 35, accessed on 22 May 2023) [84]. Additional interaction data were obtained from CORUM (accessed on 3 March 2023) [85] and RegPhos 2.0 (accessed on 24 May 2023) [76].

### 4.6. Data Visualisation

R/Bioconductor package trackViewer (https://doi.org/10.1038/s41592-019-0430-y, accessed on 20 March 2026) is used for lollipop plot data representation. The cumulative distribution of phosphosites across the quantitative differential profile datasets was visualised using the Python packages Matplotlib (version 3.10.6) and Pandas (version 3.0.1). Cytoscape (version 3.10.3) is used to visualize the upstream kinase interaction network [86] and is used to plot the downstream substrate associations.

## 5. Conclusions

In conclusion, our phosphoproteomic analysis reveals the paralog-specific co-phosphoregulatory networks that contribute to the functional divergence of TLK1 and TLK2, despite their high sequence similarity. By examining key phosphosites along with their upstream kinases, downstream substrates, and interacting proteins, we show that TLK1 is mainly involved in DNA damage checkpoint signaling and repair coordination, whereas TLK2 plays a greater role in chromatin dynamics and replication fork integrity during S-phase. These differences help explain their overlapping yet non-redundant functions in maintaining genome stability, as observed in depletion studies and disease contexts. The strong association of TLK2 with CDK-mediated regulation links its activity to cell-cycle progression, providing a mechanistic basis for its distinct roles in placental development and certain neurodegenerative conditions. Overall, this study provides a useful framework for understanding how TLK1 and TLK2 differ in function and serves as hypothesis-generating work that identifies potential regulatory relationships for further investigation. It also suggests potential therapeutic opportunities in cancers with TLK amplifications or mutations, for example, by modulating TLK2 to sensitize BRCA1-deficient tumors to PARP inhibitors, or targeting TLK2 in neurodevelopmental disorders. While growing evidence of TLK1 mutations in related conditions highlights the role of both paralogs in disease contexts. Future studies should validate these networks using site-specific perturbations and extend the analysis to isoform-level regulation to uncover further layers of control.

### Limitations of This Study

While this study provides a comprehensive analysis, certain limitations should be considered. The findings are derived from large scale integration of publicly available phosphoproteomic datasets, which despite being extensive, do not provide direct mechanisms. In addition, differences in experimental conditions, datasets composition, and analysis across studies may influence phosphosite detection and inferred co-phosphoregulation patterns. The co-phosphoregulation approach identifies coordinated phosphorylation events but does not establish direct mechanistic relationships. Likewise, the upstream kinases, substrates, and interaction networks described here are largely based on predictions and database information, and require validation in biological systems. The absence of upstream kinases for TLK1 in our analysis may also reflect limited data coverage rather than indicating a lack of biological regulation. Important regulatory mechanisms such as autophosphorylation, or context dependent signaling may not be fully captured. In this context, our study establishes a systematic framework to prioritize key phosphosites and their associated regulatory networks for TLK1 and TLK2, primarily serving as a hypothesis-generating resource. Future work should focus on targeted experimental validation, kinase-substrate assays to confirm regulatory relationships, and interaction and functional studies to define their roles in DNA damage response and chromatin regulation. Collectively, such approaches will be essential to validate these computational predictions and clarify the biological significance of TLK mediated phosphorylation networks.

## Figures and Tables

**Table 1:** Physicochemical characteristics of TLK1 and TLK2.

Category	Parameter	TLK1	TLK2
Primary structure	No. of amino acid	766	772
	Molecular weight	86,699.65	87,661.01
Domain architecture	Protein kinase	Present (456–734)	Present (462–741)
	Disordered region	1–197 346–383	24–126 180–208 342–385
Charge and stability	Theoretical pI	8.88	8.65
	Instability index	52.23	54.01

## Data Availability

All data generated or analysed during this study are included in this published article and its Supplementary Information Files.

## Supplementary Materials

The following supporting information can be downloaded at: https://www.mdpi.com/article/10.3390/ijms27125572/s1.
